# Transcriptome Analysis by RNA Sequencing of Mouse Embryonic Stem Cells Stocked on International Space Station for 1584 Days in Frozen State after Culture on the Ground

**DOI:** 10.3390/ijms25063283

**Published:** 2024-03-14

**Authors:** Kayo Yoshida, Megumi Hada, Masami Hayashi, Akane Kizu, Kohei Kitada, Kiyomi Eguchi-Kasai, Toshiaki Kokubo, Takeshi Teramura, Hiromi Hashizume Suzuki, Hitomi Watanabe, Gen Kondoh, Aiko Nagamatsu, Premkumar Saganti, Masafumi Muratani, Francis A. Cucinotta, Takashi Morita

**Affiliations:** 1 Graduate School of Medicine, Osaka Metropolitan University, Osaka 545-8585, Japankizu.akane@aijinkai-group.com (A.K.); kkitada@omu.ac.jp (K.K.); 2 Radiation Institute for Science and Engineering, Prairie View A&M University, Prairie View, TX 77446, USA; mehada@pvamu.edu (M.H.); pbsdaganti@pvamu.edu (P.S.); 3 QST National Institute of Radiation Sciences (NIRS), Chiba 263-0024, Japan; kasai.kiyomi@qst.go.jp (K.E.-K.); kokubo.toshiaki@qst.go.jp (T.K.); 4 Faculty of Medicine, Kindai University, Osaka 577-8502, Japan; teramura@med.kindai.ac.jp; 5 Japan Space Forum (JSF), Tokyo 101-0062, Japan; hashizume@jsforum.or.jp; 6 Institute for Frontier Medical Sciences, Kyoto University, Kyoto 606-8501, Japan; watanabe@infront.kyoto-u.ac.jp (H.W.); kondohg@infront.kyoto-u.ac.jp (G.K.); 7 Japan Aerospace Exploration Agency (JAXA), Tsukuba 305-8505, Japan; nagamatsu.aiko@jaxa.jp; 8 Department of Genome Biology, Faculty of Medicine, University of Tsukuba, Tsukuba 305-8575, Japan; muratani@md.tsukuba.ac.jp; 9 Department of Health Physics and Diagnostic Sciences, University of Nevada, Las Vegas, NV 89154, USA; francis.cucinotta@unlv.edu

**Keywords:** International Space Station, space radiation, mouse ES cells, RNA sequencing, gene expression, p53-related genes

## Abstract

As a space project, in “Stem Cells” by the Japan Aerospace Exploration Agency (JAXA), frozen mouse ES cells were stored on the International Space Station (ISS) in the Minus Eighty Degree Laboratory Freezer for ISS (MELFI) for 1584 days. After taking these cells back to the ground, the cells were thawed and cultured, and their gene expressions were comprehensively analyzed using RNA sequencing in order to elucidate the early response of the cells to long-time exposure to space radiation consisting of various ionized particles. The comparisons of gene expression involved in double-stranded break (DSB) repair were examined. The expressions of most of the genes that were involved in homologous recombination (HR) and non-homologous end joining (NHEJ) were not significantly changed between the ISS-stocked cells and ground-stocked control cells. However, the transcription of Trp53inp1 (tumor protein 53 induced nuclear protein-1), Cdkn1a (p21), and Mdm2 genes increased in ISS-stocked cells as well as Fe ion-irradiated cells compared to control cells. This suggests that accumulated DNA damage caused by space radiation exposure would activate these genes, which are involved in cell cycle arrest for repair and apoptosis in a p53-dependent or -independent manner, in order to prevent cells with damaged genomes from proliferating and forming tumors.

## 1. Introduction

Considering long-term manned spaceflight missions, such as lunar surface exploration or Mars exploration, the possibility of astronauts being harmed by space radiation like heavy ion particles becomes a very important issue [1,2,3,4,5]. In space outside of the atmosphere of the Earth, high-energy particles are the major constituents of the galactic cosmic rays. The trapped particles of protons and electrons are surrounded as inner and outer Van Allen belts, respectively, by the magnetic field of the Earth. During solar particle events, electrons and protons and some heavy ions, such as C ions and Fe ions, are emitted due to the activity of the surface of the sun, with energies up to a few hundred MeV/n. In contrast, the galactic cosmic rays contain many types of ions, such as electrons, protons, and many types of heavier ion particles, with energies of many 1000 s of MeV/n. When we think of the human flight to and stay in space, the effects of protons and heavy ions are suggested to have severe effects on the human body including cancer, circulatory disease, and cognitive risks [1,6,7,8,9,10] that must be properly assessed.

**Figure 1:**
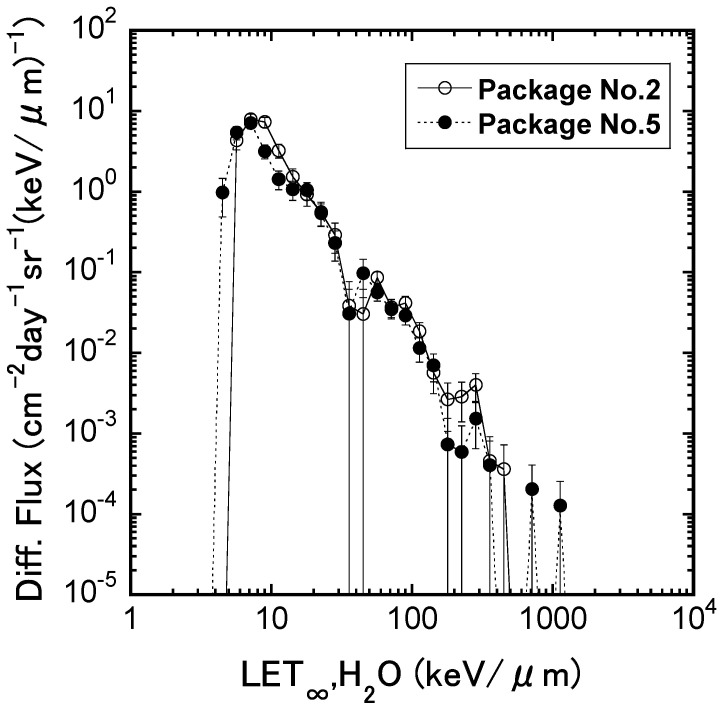
Differential particle flux function of LET_∞_, H_2_O measured with CR-39 plastic detector, and PNTDs in a flight of ISS for 1584 days. LET distribution was obtained by subtracting background LET distribution, measured by ground control.

The dose-equivalent rates, indicating the biological effect that is calculated from a physical dose measurement, are about 0.5–0.62 mSv/day on the International Space Station (ISS) [11,12], 1.15 mSv/day on the surface of the moon of the Earth [13,14], 0.5 mSv/day on the surface of Mars [15], and 1.84 mSv/day during the flight from the Earth to Mars [16,17,18]. In our “Stem Cells” experiment, mouse ES cells were frozen and kept in MELFI on the ISS for 1584 days to quantitate the biological effect of space radiation [18,19]. The physical doses were measured by a passive dosimeter for life science experiments in space (PADLES), and it was revealed that the absorbed dose rate of space radiation in MELFI was 0.36 mGy/day, and the dose-equivalent rate was 0.53 mSv/day, as shown in Figure 1 [19]. About 4.1% of particles had more than 10 keV/μm of LET (linear energy transfer), but they contributed 35.3% of total dose-equivalent. The quality factor of the ISS was estimated to be about 1.48 on the ISS. This physically estimated value was similar to the relative biological effectiveness (RBE) of 1.54, which was established by the biological measurement of chromosome aberration using frozen mouse ES cells in the MELFI freezer on the ISS [19].

The dose-equivalent rate of space radiation on the ISS is about 100 times higher and about 300 times higher that during the traveling pathway compared with the surface of the Earth (0.006 mSv/day), and the content of heavy ion is also high compared to the ground control. Therefore, we examined the responses of cells against space radiation sources composed of various species of particles with various energies or LETs, resulting in exploration risks [20]. As such conditions could not be achieved on the ground, we launched mouse ES cells in a frozen state for 1584 days, and after taking them back to the ground and thawing and culturing them, we comprehensively examined the expression of genes responding to space radiation stresses compared with the ground-based backup controls by RNA-sequencing analysis. We also compared the effect of the loss of DNA repair of the gene histone H2AX to the transcriptome between the ISS and the ground control. This experiment reflects the acute response of the cells to the chronically accumulated damage by space radiation (totaling 830 mSv during 1584 days) after thawing.

The purpose of our research is to comprehensively compare the gene expression profiles of cells on the ISS and the ground control (BU) to identify the genes that respond to DNA damage caused by space radiation and those that regulate their expression. The complex DNA damage caused by space radiation may induce a novel gene expression for repair. The damage may also enhance the expression of previously unpredicted genes for apoptosis. If such genes are identified, they may become good markers of DNA damage that is specific to space radiation. In addition, we may be able to identify a gene expression related to non-DNA targeted effects, which may lead to genomic instability and cancer, as well as the effects resulting from direct DNA damage by low-dose radiation. The targeted and non-targeted effects may have a potential influence on human health risks, so that the transcriptome analyses of cells that are exposed to space radiation may contribute to the risk assessment.

## 2. Results

### 2.1. The Irradiation Effects of Fe Ions on the Mouse ES Cells in terms of Transcriptome Profile

**Figure 2:**
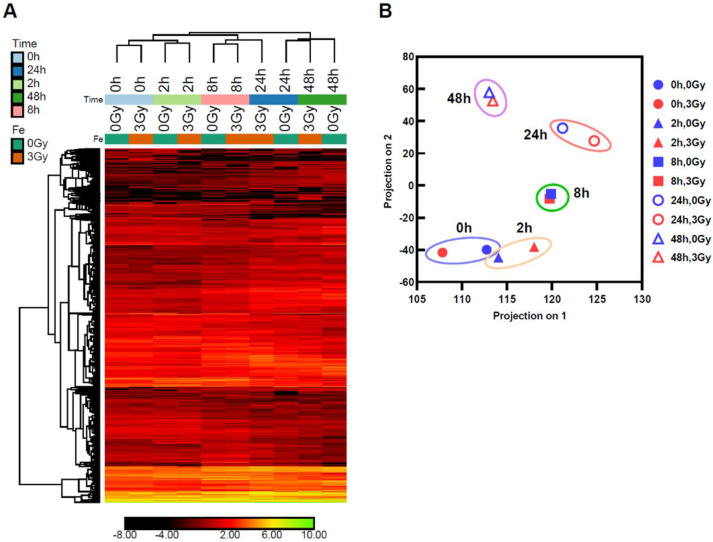
Filtering of genes altered by Fe ion irradiation. (**A**) Heatmap of 2924 genes across time course. (**B**) PCA plot based on filtered genes. The data of the same culture time group are shown in a circle.

To investigate the irradiation effects on mouse ES cells of ionizing radiation, we irradiated frozen wild-type mouse ES cells, using an Fe ion of 3 Gy (500 MeV/n and LET 218 keV/μm) as a reference. The frozen wild-type mouse ES cells with or without Fe ion irradiation were thawed and cultured for 0, 2, 8, 24, or 48 h in order to examine the expression profile over time. RNA was extracted and analyzed by RNA sequencing. The expression of 51,751 kinds of gene sequences were quantified and normalized across the ten samples. The 2924 genes were selected (|fold change| > 3, |difference| > 1) and are shown in Figure 2A. The Principal Component Analysis (PCA) plot based on the filtered genes (Figure 2B) indicated that the ES cell culture showed a time-dependent transition of its RNA expression patterns.

**Figure 3:**
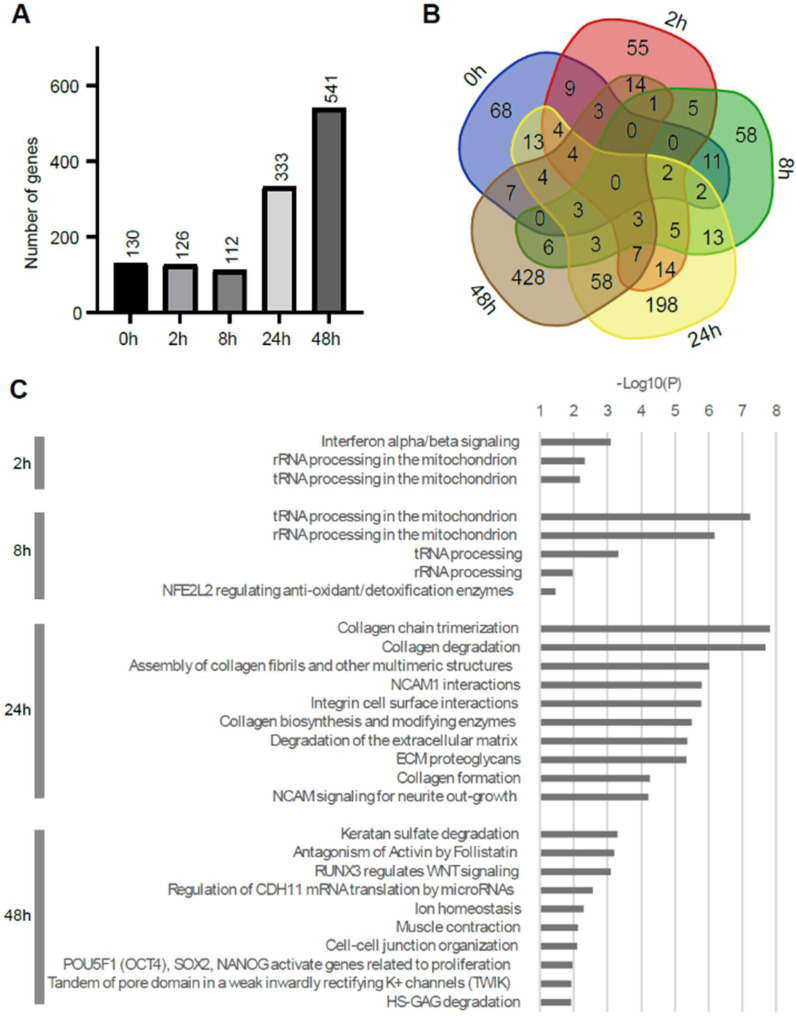
Pairwise analysis between Fe ion-irradiated and control samples. (**A**) Number of genes altered in Fe ion-irradiated samples at each time point. (**B**) Venn diagram comparing genes identified by pairwise comparisons. (**C**) Reactome pathways enriched in each list of genes across time course. Reactome pathways with *p*-value < 0.05 are shown for 2 and 8 h time points. There was no enriched term at 0 h. Only top 10 pathways are listed for 24 and 48 h time points.

**Figure 4:**
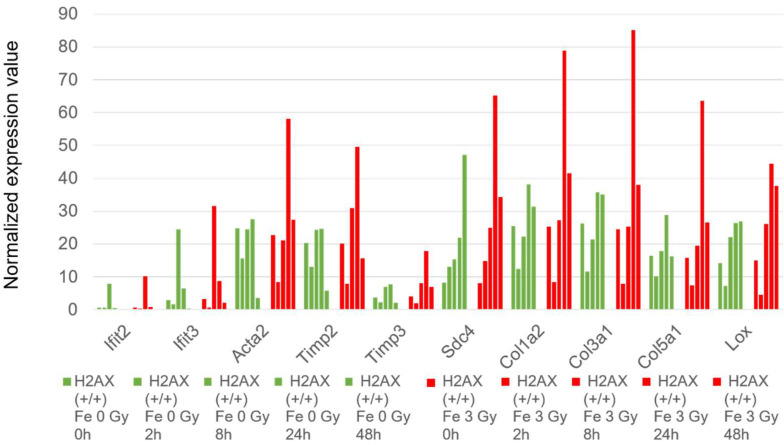
A comparison of temporal changes in the gene expressions of wild-type mouse ES cells. The relative amount of RNA of the respective genes in wild-type mouse ES cells are represented. The values of cells without irradiation are shown in green, and those of the cells that are irradiated with 3 Gy of Fe ions are shown in red. They are arranged chronologically as 0, 2, 8, 24, and 48 h from right to left. The vertical axis shows the normalized expression values.

To examine the potential impact of Fe ion irradiation, pairwise comparisons of the cells between Fe ion-irradiated and unirradiated cells were performed. Figure 3A shows the number of genes that was selected by filtering |fc| > 2 and |difference| > 1 (difference meant that the differences between expression levels of genes were more than 1 in order to exclude the small expressions with large folds) at each time point. This result indicated that the incubation time of ES cells had an impact in the early and late phases, with increasingly altered genes towards 24 and 48 h post inoculation. The Venn diagram in Figure 3B supports that distinct groups of genes were altered at each time point. A pathway analysis using the Reactome tool accessed on 13 January 2024 (https://reactome.org/) showed that the interferon signaling was a unique enriched biological process at the 2 h time point (Figure 3C). This enrichment was supported by Ifit2 and Ifit3 genes [21], which are induced by interferon alpha. At the 2 and 8 h time points, mitochondrial genes were also enriched, indicating the activity of the beginning of cell physiology by mitochondrial function or the increase in the copy number of mitochondrial DNA. At the 24–48 h time points, the cell cycle of the ES cells was completed approximately three or more times, because the cell cycle time of the ES cells was found to be 13 to 15 h. The prominent increasing expression of genes at 24–48 h were detected for Col1a1, Acta2 (actin assembly-inducing protein), Timp2, Timp3, Sdc4, Col1a2, Col3a1, Col5a1, and Lox (Collagen crosslinking) genes. Collagens are extracellular matrix proteins and the Timp protein is related to the degradation of the extracellular matrix and the Sdc4 gene codes for transmembrane heparan sulfate proteoglycan [22]. They were thought to be necessary for contact between cells or cells and the surface before cell differentiation, such as trophectoderm [23]. Their expressions were enhanced by Fe ion irradiation, indicating the early differentiation that is induced by radiation (Figure 4).

**Figure 5:**
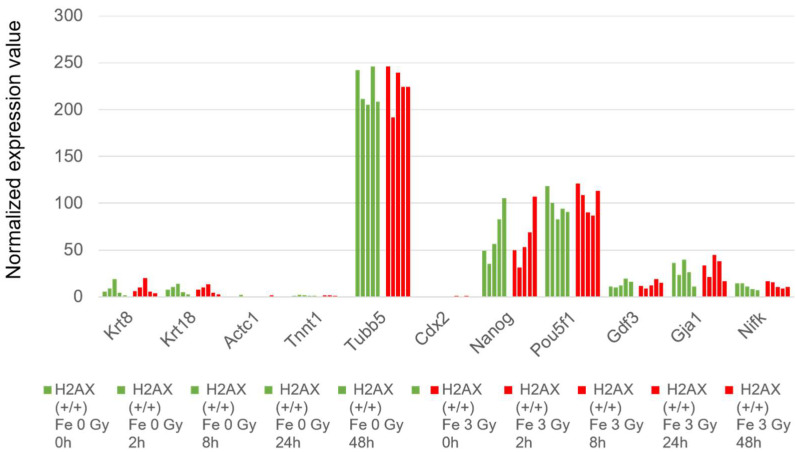
Temporal changes in the gene expression of wild-type mouse ES cells related to differentiation. The normalized value of the expression of the respective gene in wild-type mouse ES cells are demonstrated. The expression values of cells without irradiation are shown in green, and those irradiated with 3 Gy of Fe ion are shown in red. They are arranged chronologically as 0, 2, 8, 24, and 48 h from right to left. The vertical axis shows the normalized expression values.

However, as shown in Figure 5, at 48 h after culture of the cells, we did not detect an apparent increase in the transcription of differentiation marker genes like keratin genes (Krt8, Krt18) [24,25], actin-related genes (Actc, Tnnt1), the Tubulin gene (Tubb5), which is necessary for differentiation to endoderm, and the Cdx2 gene for trophectoderm formation [26] in the ES cells that were irradiated by Fe ions. Consistently, the cells that were irradiated with Fe ions maintained the expression of undifferentiation marker genes such as Nanog, Pou5f1 (Oct4), GDF3 (Growth differentiation factor), GJA (Gap junction protein), and the Nifk (Mki67ip) gene, which promotes ES cell self-renewal, suggesting that the ES cells were not differentiated to endoderm or trophectoderm by irradiation [27,28].

### 2.2. The Comparison of the Expression Profile of the Transcriptome between Mouse ES Cells of Ground Backup Control and ISS-Stocked Cells

We launched wild-type and radio-sensitized histone H2AX-homozygously deficient mouse ES cells to the ISS and stocked the frozen cells in the MELFI freezer for 1584 days. In order to analyze the response of cells by the transcription of the genes of ES cells whose DNA accumulated following damage from space radiation, we cultured the cells for 2 and 8 h to search for an early response to damage signaling and repair. It was found that many genes that were involved in DNA repair and cell cycle and stress responses worked within 30 min or several hours after exposure [29]. The absorbed dose of the cells in the MELFI freezer was 0.56 Gy after 1584 days, and the calculated equivalent dose was 0.83 Sv after 1584 days using quality factors of ICRP60 [30]. Thus, the dose of irradiation on the ISS was low compared with the Fe ion (3 Gy, 60 Sv) irradiation experiment, as mentioned above.

**Figure 6:**
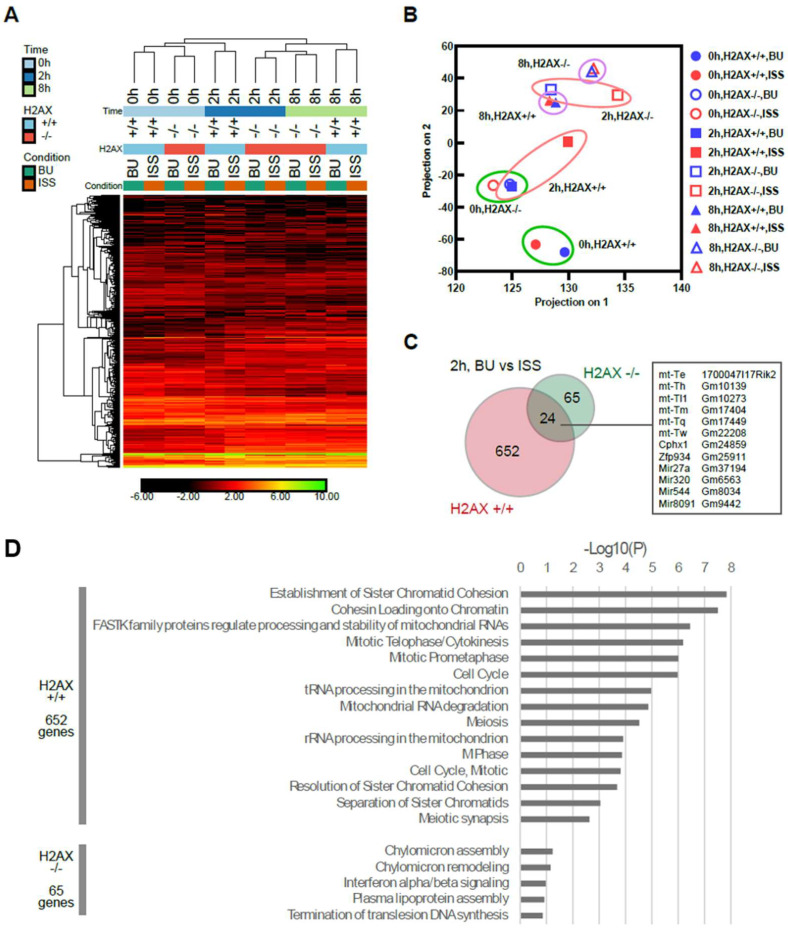
RNA sequencing results from flight experiment. (**A**) Heatmap showing 3365 genes differentially expressed across 12 samples. (**B**) PCA plot showing post-filtering gene profiles. (**C**) Venn diagram comparing 2 h samples between the presence and absence of H2AX. The data of the same culture time and the same genotype of histone H2AX are circled. (**D**) Reactome pathway analysis results for genes shown in panel C at 2 h time points.

Overall, the ES cell identities were maintained in all the samples. However, the heatmap of the selected 3365 genes (|fc| > 3, |difference| > 1, across 12 samples) indicated differences between the backup control and the ISS-stocked samples of each genotype “”(histone H2AX +/+ or −/−) or incubation conditions (Figure 6A). To evaluate the trend of the RNA expression profiles, a PCA plot was produced (Figure 6B). It revealed that the difference between the ground backup controls (BU) and the ISS appeared at the 2 h time point, but not clearly at the 0 or 8 h time points. When the BU and ISS samples were compared by pairwise analysis (|fc| > 2, |difference| > 1), 652 genes were detected. Most of them were H2AX-dependent, 65 genes were unique to the H2AX −/− cells, and 24 genes were common (Figure 6C). H2AX-dependent genes were mainly cell-cycle-related genes (Figure 6D), which might be caused by DNA instability. Common differences included RNAs from mitochondrial genes. There was no enriched pathway (*p* < 0.05) for the 65 genes that were unique to the H2AX −/− cells, which might compensate for the defects in the H2AX gene.

Subsequently, the results were presented as the expression pattern of cells on the ISS and BU, and the expression ratios for the ISS were compared to those of the BU controls. We selected the genes that were involved in double-stranded DNA break repair genes [31], the cell cycle regulation, and the p53 pathway for RNA sequencing analyses. In addition, we carried out transcriptome analysis of the H2AX-homozygously deficient mouse ES cells on the ISS.

### 2.3. Homologous Recombination Repair Genes

The homologous recombination repairs the double-stranded DNA break caused by the ionizing radiation, UV, or reactive oxygen species (ROS) in the S to G2/M phases. It begins with the Atm or Atr signaling caused by the DNA damage [29], followed by phosphorylation of Chk2, or Chk1 kinases, respectively [32]. They lead to the processing of the broken ends of DNA by an MRN complex consisting of Mre11, Rad50, and Nbs1 proteins; an ExoI/BLM complex; and CtIp protein [33,34]. The recessed single-strand DNA is stabilized by RPA replication protein A (RPA). The TopBP1 protein also colocalizes with ionizing radiation-induced single-stranded DNA foci with Rad50, Brca1, Atm, and Blm proteins [35,36,37]. The recombination proteins were loaded onto the processed single-stranded DNA by Brca1 and Brca2 proteins. The recombination proteins, such as Rad 51, 52, and 54 proteins and Rad51-related proteins (Xrcc2 and 3), catalyze the searching of homologous DNA ends and strand exchanges [38].

**Figure 7:**
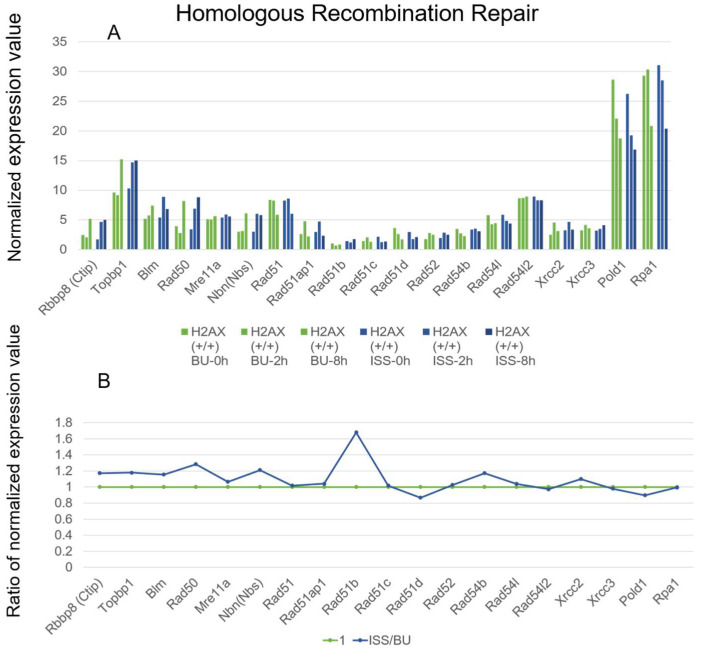
The expressions of genes involved in homologous recombination repair in wild-type mouse ES cells on the ISS and on the ground (**A**) The RNA levels of the respective genes in wild-type mouse ES cells are depicted. Blue bars represent the values of cells stocked on the ISS (ISS), while green bars represent the ground backup controls (BU). They are arranged chronologically as 0, 2, and 8 h after culture of the cells from right to left. The vertical axis displays the normalized expression values. (**B**) For each gene, the normalized expression values of culture times 0, 2, and 8 h were summed, and the ratio of the total ISS value to the total BU value was calculated. The BU value is shown as 1 in green, and the ratio of the ISS value is shown in blue.

From the RNA sequencing analysis, the expression patterns of most genes that were involved in homologous recombination were not changed between the ISS-stocked ES cells and the ground control cells (Figure 7). The figures show that genes whose expression increased on the ISS also increased on the ground, and genes whose expression decreased on the ISS also decreased on the ground, making it difficult to compare the expression patterns. We considered not only individual changes over time (panel A), but also the summed-up values over the initial period (0–8 h) in panel B were important. We also took into account whether the sum of the expression levels was very small or not. The gene expression of the Rad51b gene was increased 1.7-fold in the ISS-stocked cells compared to the BU controls after 2–8 h of culture, but the expression level was very low. The expression level of the Rad51 gene, which was pivotal for homologous recombination [39,40,41], remained constant during 0–8 h of culture and even after exposure to space radiation on the ISS. 

### 2.4. Non-Homologous End Joining Repair Genes

We analyzed the expression of genes involved in classical non-homologous end joining repair for double-stranded DNA breaks. In this process, the heterodimers of Xrcc6 (Ku70) and Xrcc5 (Ku80) proteins attach to the broken DNA ends, and DNA-PKcs protein, activated by phosphorylation, binds to the broken sites [42]. Artemis nucleases that are phosphorylated by Atm or ATR by ionizing radiation (IR) or UV irradiation, respectively, excise the bases at the broken ends [43]. The rejoining of the DNA ends is carried out by Xrcc4 and DNA Ligase4 proteins [39]. The Artemis proteins are required for DNA-damage-induced G2/M cell cycle arrest [44].

**Figure 8:**
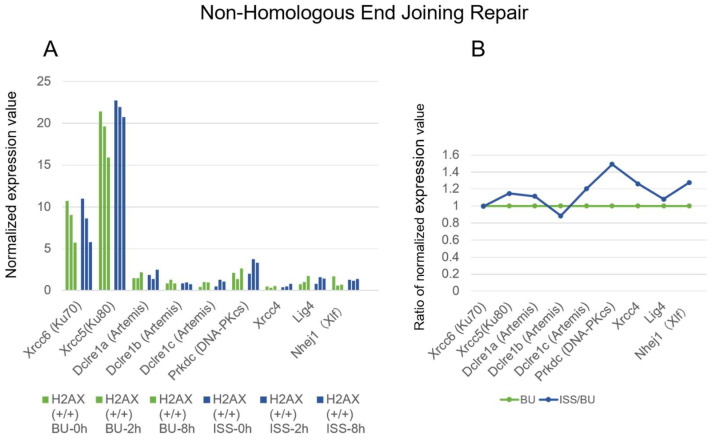
The expressions of genes involved in non-homologous end joining repair in wild-type mouse ES cells on the ISS and on the ground. (**A**) The RNA levels of the respective genes in wild-type mouse ES cells are depicted. Blue bars represent the values of cells stocked on the ISS (ISS), while green bars represent the BU. They are arranged chronologically as 0, 2, and 8 h after culture of cells from right to left. The vertical axis displays the normalized expression values. (**B**) For each gene, the normalized expression values of culture times of 0, 2, and 8 h were summed, and the ratio of the total ISS value to the total BU value was calculated. The BU value is shown as 1 in green, and the ratio of the ISS value is shown in blue.

As shown in Figure 8, Ku70 and Ku80 transcripts were abundant and slightly decreased after culture, but differences in expressions were not detected between the ISS and BU groups. The DNA-PKcs gene expression was increased (~1.5-fold) in the ISS cells compared with the BU, but the expression level was very low.

### 2.5. Cell Cycle Regulation and p53 Pathways Genes

**Figure 9:**
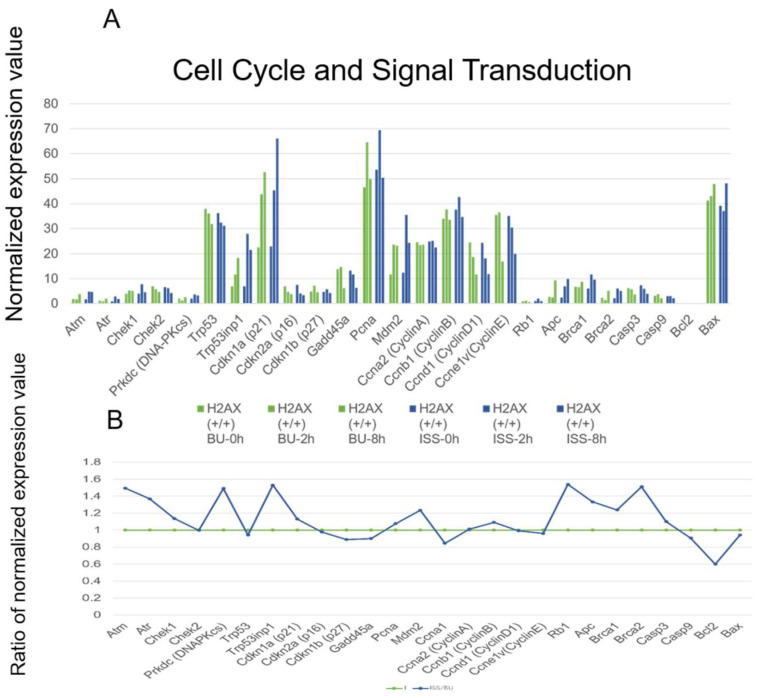
The expressions of genes involved in cell cycle and signal transduction in wild-type mouse ES cells on the ISS and on the ground. (**A**) The RNA levels of the respective genes in wild-type mouse ES cells are depicted. Blue bars represent the values of cells stocked on the ISS (ISS), while green bars represent the BU. They are arranged chronologically as 0, 2, and 8 h after culture of cells from right to left. The vertical axis displays the normalized expression values. (**B**) For each gene, the normalized expression values of culture times of 0, 2, and 8 h were summed, and the ratio of the total ISS value to the total BU value was calculated. The BU value is shown as 1 in green, and the ratio of the ISS value is shown in blue.

The cell cycle is regulated by cyclins of A, B, D, and E; cyclin-dependent kinases Cdk1, 2, 4, and 6; and cyclin-dependent kinase inhibitor (Cdkn) genes. In this experiment, the cyclin A, B, D, E genes were moderately expressed, and their expressions were not changed by being stocked on the ISS compared to the backup control, as shown in Figure 9. The cyclin-dependent kinase genes, including Cdk1, Cdk2, Cdk4, and Cdk5, were also moderately expressed, but the Cdk6 expression level was low. The expressions of these genes were not affected by the culture time of the cells or being stocked in space and on ground.

However, when cells are exposed to radiation or chemical stresses, the p53 pathway begins to function. DNA damage activates ATM or ATR and phosphorylates Chk2 or Chk1 proteins, respectively. They subsequently phosphorylate Cdc25C to arrest the cells at the G2 checkpoint. They also phosphorylate the p53 protein to make it stable in the nuclei. The phosphorylated p53 protein induces transcription of Cdkn1a (p21), Mdm2, and Trp53inp1 [45,46,47], resulting in G1/S or G2/M arrest of the cell cycle and, ultimately, cellular senescence or apoptosis. The Bcl-2 protein inhibits cells from going into apoptosis, but BAX and BAK proteins change the outer mitochondrial membrane’s permeability and promote CytochromeC excretion, resulting in the activation of Casp3 and apoptosis [48,49].

In this experiment, the expression levels of Atm, Atr, Rb1, APC, and Brca2 genes were increased about 1.4-fold in the ISS stock compared with the BU, but their expression levels were very low. The expression of DNA-PKcs, whose product was thought to phosphorylate p53, was increased by being stocked on the ISS compared with the BU, but the expression level was very low. The level of the p53 transcript was high, but it was not increased by being stocked on the ISS. The expression of the Trp53inp1 gene was also high and showed an increase in the ISS-stocked group by about 1.53-fold compared with the sums of the normalized expression values after 0, 2, 8 h. The transcription of the Cdkn1a (p21) gene was high compared with the other cyclin-dependent kinase inhibitors, Cdkn2a (p16) and Cdkn1b (p27). Further, the p21 gene expression was increased by being stocked on the ISS by about 1.13-fold compared to the backup control. The expression of Mdm2 was high, and the level was increased by being stocked on the ISS by about 1.23-fold.

The Rb1 protein binds to transcription factor E2F and regulates G1/S or G2/M arrest in differentiated ES cells [50,51]. Its interaction to E2F is also involved in the homologous recombination repair of DSBs [52]. Although the expression of the Rb and Brca2 genes were increased by being stocked on the ISS, their expression levels were very low.

**Figure 10:**
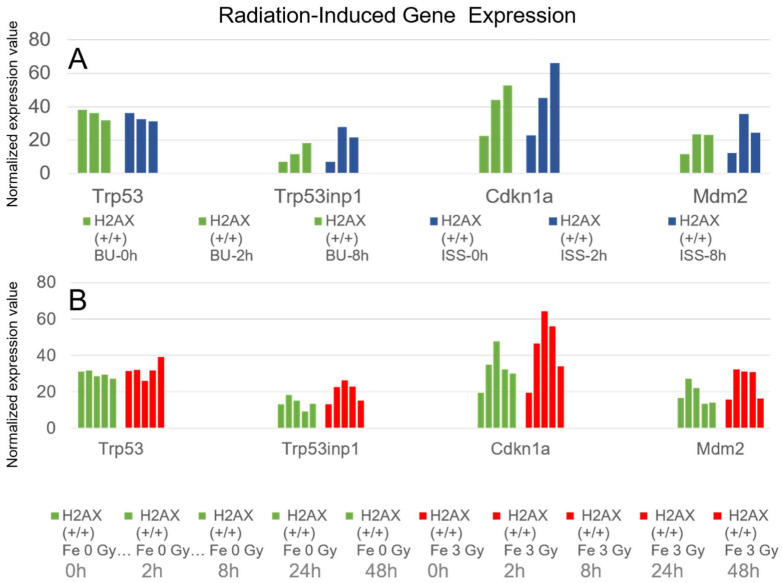
Enhancement of gene expression by ionizing radiation in three genes (Trp53inp1, p21, and Mdm2); (**A**) transcriptional enhancement was observed by ISS preservation for 1584 days compared to ground backup control using wild-type mouse ES cells (129/C57BL/6). Green bar is BU, and blue bar is ISS-stocked cells. They are arranged chronologically as 0, 2, and 8 h after culture of cells from right to left. (**B**) Although the transcription was not changed for Trp53 gene, expression of genes (Trp53inp1, p21, and Mdm2) increased by irradiation with Fe ions at a dose of 3.0 Gy using an accelerator using wild-type mouse ES cells (B6-G). Green bar represents unirradiated cells, and red bar represents Fe ion-irradiated cells. The bars are arranged chronologically as 0, 2, 8, 24, and 48 h from right to left. Vertical axis shows normalized expression values.

We focused more on genes whose expression increased due to the effects of space radiation on the ISS. Although the changes in gene expression between the ISS and BU groups were small for most of the genes, we identified only three genes whose expressions were enhanced by radiation, even if slightly, after comparing diagrams created from the 51,751 gene sequencing data. The three genes were Trp53inp1, p21, and Mdm2, whose transcriptions were known to be enhanced by activated p53 protein. The Trp53inp1 gene expression is induced by stresses, as well as ionizing radiation [53,54]. We investigated whether the expression of these genes was enhanced not only by being stocked on the ISS, but also by irradiation with Fe ion beams. Although the absorbed doses were different between the ISS (0.56 mGy) and Fe ions (3.0 Gy), the expression of these genes was found to be enhanced when they were irradiated with Fe ion beams, as shown in Figure 10 and Table 1. Consequently, it became evident that p53-induced genes responded to exposure to space radiation on the ISS.

For the ES cells that were preserved on the ISS and on the ground, the normalized expression values of each gene after 0, 2, and 8 h of culture were totaled, and the ratio was displayed. For the ES cells that were irradiated with Fe ion beams and those that were not irradiated, the normalized gene expression values of each gene after 0, 2, and 8 h and after 0, 2, 8, 24, and 48 h were totaled, respectively. They were compared between irradiated and unirradiated, and the ratios were calculated and are displayed in the table.

### 2.6. Histone H2AX-Dependent Gene Expression

**Figure 11:**
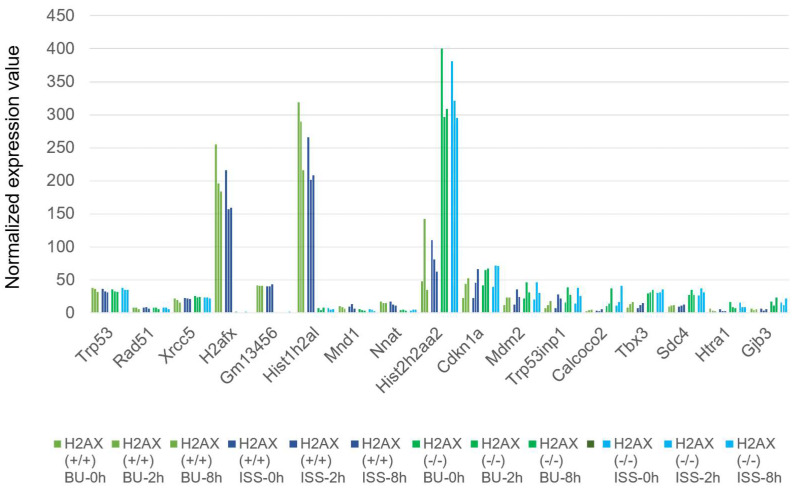
The expressions of genes that have been enhanced or attenuated by space radiation in wild-type and histone H2AX-deficient mouse ES cells both on the ISS and on the ground. The normalized expression values of the respective genes in wild-type mouse ES cells are depicted. Blue bars represent the values of cells stocked on the ISS, while green bars represent the BU. They are arranged chronologically as 0, 2, and 8 h after culture of cells from right to left. The normalized expression values of the respective genes in histone H2AX-deficient mouse ES cells are depicted for both ISS-stocked cells (light blue) and ground-stocked cells (bright green). They are also arranged chronologically as 0, 2, and 8 h after culture of cells from right to left. The vertical axis displays the normalized expression values.

We analyzed the gene expression of histone H2AX-homozygously deficient ES cells on the ISS. The histone H2AX gene is involved in DNA damage repair, and the deficient ES cells became more sensitive to ionizing radiation than the wild-type cells did [55,56,57]. The expression patterns of most genes, such as the Trp53 (×0.94), Rad51 (×0.96), and Xrcc5 (×1.28) genes, were not changed by the lack of H2AX when comparing the sum of the 0-, 2-, and 8-hour expression values to the ground backup controls (Figure 11). However, we observed a significantly decreased expression of pseudogenes and Gm13456 (eukaryotic translation elongation factor 1 alpha1 pseudogene; ×0.02), Hist1h2al (Histone H2A pseudogene2; ×0.02), Mnd1 (meiotic nuclear division 1; ×0.50), and Nnat (Nueronatin; ×0.27) genes. On the contrary, the expression of the Hist2h2aa2 (×1.46) gene coding for the histone H2AX protein in a cluster on chromosome 3 was significantly increased by the histone H2AX gene deficiency. This gene is known to be transcribed using anti-sense DNA [58]. The expressions of the p21 (×1.28), Mdm2 (×1.69), and Trp53inp1 (×2.20) genes were slightly increased by the H2AX deficiency. The transcriptions of the other genes like the Calcoco2 (calcium-binding and coiled-coil domain 2; × 5.29), Tbx3 (T-box transcription factor 3; ×2.50), Sdc4 (Syndecan4; ×2.76), Htra1 (HtrA serine peptidase 1; ×2.66), and Gjb3 (Gap junction protein beta3; ×3.19) genes were also increased by the H2AX deletion.

## 3. Discussion

### 3.1. The Transcriptome of Mouse ES Cells on the ISS

As the ground-based experiment of transcriptome, the modulation of tumor progression by age and irradiation (proton and Fe ion) was reported [59,60]. Also, transcriptome analysis was carried out on the ground using low-dose irradiation and modeled microgravity showing multiple altered neurological pathways [61] or a hematological system with transforming growth factor β signaling [62]. During the flight on the ISS for 10 days, a decrease in the expression of the newt (*Pleurodeles waltl*) gene of DNA repair (Pol mu) was observed due to the space radiation or microgravity [63]. Comprehensive analyses of transcripts have been carried out on the International Space Station to examine the effects of space radiation and microgravity on animals, and it was found that the expression of several microRNAs in blood might regulate the TGFβ1 gene, which is a master regulator coordinating for a systemic response to microgravity and space radiation [64]. The gene expression of circadian rhythm-related genes was altered on the ISS compared with those in the ground control [65]. Mouse heart cells flown on the ISS for 30 days showed an absence of cellular senescence and significant upregulation of transcripts associated with the cell cycle [66]. In the 13-day mission in space, mice were onboard, and their lung tissues were analyzed by RNA sequencing. The results showed the increased gene expression of an anti-adhesive gene, Spock1 [67]. In addition, from the big data from many animal experiments during spaceflight and the data from NASA’s astronauts and twin studies, stress on mitochondria was revealed to be a consistent phenotype of spaceflight [68,69].

In this experiment, we analyzed the transcriptome of the ISS-stocked cells, irradiated by space radiation, for 1584 days. A significant difference in the transcriptome was not found between the ISS-stocked cells and the ground control. This might be due to the low absorbed dose of space radiation (0.56 Gy/1584 days). Furthermore, no clear enhancement of gene expression was observed for homologous recombination repair and non-homologous end joining repair. We also examined DNA replication genes (Figure S1), mismatch repair genes (Figure S2), base excision repair genes (Figure S3), nucleotide excision repair genes (Figure S4), and mitochondrial genes (Figure S5). However, we did not detect a significant enhancement or repression of the expression between the ISS and backup cells (BU). Therefore, in mammals, the rapid response to ionizing radiation may be supported by modifications of repair proteins such as phosphorylation or by degradation of the proteins after ubiquitination. The induction of gene expression may not be suited for early responses. Although we did not find changes in gene expression in undifferentiated mouse ES cells, the other differentiated cells, forming tissues or organs, may show various profiles of transcriptomes by space radiation. In this experiment, we found that the amounts of transcripts of Ku70 and Ku80 proteins in non-homologous end joining and those for glycosylases that are involved in base excision repairs were relatively abundant, presumably for the immediate recognition of DNA damage in the cells.

### 3.2. The Enhancement of Expression in p53-Related Genes in ES Cells on the ISS

In the transcriptome analysis, it was found that the expression of the Trp53inp1, p21, and Mdm2 genes were increased by exposure to space radiation on the ISS, and this was confirmed by Fe ion irradiation, although the doses were different. Similar results from the enhancement of those gene expressions were reported from low-LET ionizing radiation [70], oxidative stress [71], inhibition of p53 degradation [47], and chemotherapy in cancer [72,73]. So, it may be assumed that the accumulated DNA damage caused by space radiation on the ISS activated the p53 protein to induce transcription of the p21, Trp53inp1, and Mdm1 genes and regulated the repair and apoptosis of DNA-damaged cells.

**Figure 12:**
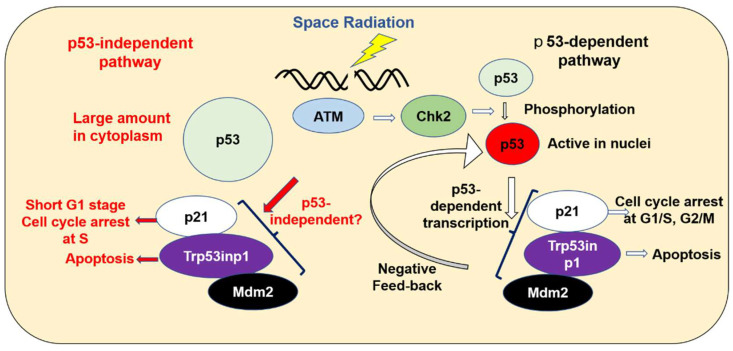
Cellular response to space radiation.

Mouse ES cells have the pluripotency to self-renew and to differentiate into all cell types. So, mutations or chromosome aberrations in ES cells caused by the radiation or stresses seriously injure the organisms [74]. The undifferentiated ES cells have a higher amount of p53 RNA in their cytoplasm, but not in their nuclei [75,76,77,78]. Furthermore, the ES cells have a shorter G1 phase, and the ionizing radiation arrests the ES cells not in the G1 phase, but in the S phase and the G2 phase [79,80]. These results indicate that the undifferentiated ES cells may have a unique pathway to repair or exclude the DNA damage with or without p53 protein [81,82,83,84] (Figure 12). The undifferentiated ES cells were reported to enhance the p21 transcription by irradiation [77], and the transcription of the Trp53inp1 gene could be promoted by E2F and p73 proteins [85]. From these, the distinct mechanism for enhancing the p53-related gene expression must be elucidated to understand the early response of undifferentiated ES cells [86] on the ISS-stocked cells that were exposed to space radiation.

The present study suggests that accumulated DNA damage caused by space radiation induces the transcription of the p21, Trp53inp1, and Mdm2 genes. In differentiated cells, the p53 protein promotes the transcription of those genes. However, undifferentiated mouse ES cells have a high amount of p53 transcripts, but they are located in the cytoplasm. It is possible that p53 can still influence the expression of the p21, Trp53inp1, and Mdm2 genes, either directly or indirectly. The increased p21 protein might arrest the cell cycle in the S phase to repair DNA damage, and the Trp53inp1 protein might be involved in the apoptosis of the irradiated ES cells.

### 3.3. The Effects of Transcriptome Caused by the Lack of the Histone H2AX Gene

We launched histone H2AX-homozygously deficient mouse ES cells onto the ISS. The deletion of the gene did not affect most of the gene expressions. The transcription of the pseudogenes of Gm13456 and Hist1h2al completely stopped, while that of the Hist2h2aa2 gene coding for clustered major histone H2A protein increased remarkably due to the lack of the H2AX gene. It may compensate for the lack of the minor histone H2AX gene. However, the major histone H2A protein did not have the longer C-terminal part that is characteristic for the histone H2AX protein containing Ser residues for phosphorylation. Thus, the major histone H2A protein cannot support the DNA repair functions of the histone H2AX protein. The gene expressions of p21, p53inp1, and Mdm2 were slightly increased by the H2AX gene deletion, suggesting that the chronic DNA instability caused by the lack of histone H2AX may induce transcription. In conclusion, the histone H2AX-homozygously deficient cells changed the transcriptome profiles of a few genes greatly, but they could not compensate for the lost function, thus resulting in a phenotype with increased radio sensitivity.

## 4. Materials and Methods

### 4.1. Mouse ES Cells

For Fe ion irradiation studies, we used mouse B6-G cells that had transduced green fluorescent proteina gene (GFP) to C57BL/6 wild-type mouse ES cells, obtained from Institute of Physical and Chemical Research (RIKEN) in Japan. For space experiments, we used wild-type mouse ES cells. Preparation of mouse ES cells lacking the histone H2AX gene with 129 Sv and C57BL/6 heterozygous background was performed as described before [19].

### 4.2. Preparation of Space Samples

After culturing the wild-type and histone H2AX gene-deficient mouse ES cells, they were suspended in a cell cryopreservation solution, CELLBANKER (Zenoaq Resource, Fukushima, Japan), dispensed into a cryotube at a concentration of 2 × 10^6^ cells/tube, and cryopreserved (−150 °C). These tubes were launched on 2 March 2013 as a “Stem Cells” project from the NASA Kennedy Space Center (Merritt Island, FL, USA) in a GLACIER freezer (−95 °C) aboard the SpaceX-2 Dragon. After arriving at the ISS, the cells were stored in a MELFI freezer (−95 °C) in the Japanese experiment module “Kibo”. The cells that were stored for the longest time were brought back from the ISS to the ground again on 3 July 2017 using Glacier (−95 °C). The samples (ISS) were transported from the Kennedy Space Center to JAXA, Tsukuba Space Center in Ibaraki, Japan, using dry ice (−79 °C). Ground-preserved control cells (BU) were stored at −95 °C in JAXA (Tukuba, Japan), Tsukuba Space Center, at exactly the same time and for the same duration and at the same temperature as the space samples. The recovered ISS and BU samples in Japan were transferred to Osaka City University on dry ice, where they were stored at −150 °C. They were analyzed about one year later. Physical dosimeters, Bio PADLES, were attached to the package of cell samples both on the ISS and on the round at JAXA, Tsukuba Space Center.

### 4.3. Irradiation by Accelerator

Using an accelerator, the Heavy Ion Medical Accelerator in Chiba (HIMAC) at QST National Institute of Radiological Sciences, mouse ES cells were irradiated by Fe ion particle beams in a frozen state on dry ice as references for single beam irradiation. The Fe ion beam was 500 MeV/n, and the LET was 218 keV/μm. When irradiating the cells with Fe ion beams using an accelerator on the ground, the cryotube (1.5 mL) containing the cells was placed in a Styrofoam box with a sufficient amount of dry ice (−79 °C) around the opposite side of the irradiation side. We put the cryotube upright and covered the top with dry ice. As the absorbed dose rate of Fe ion irradiation was about 300 mGy/min, it took about 10 min for irradiation of 3 Gy (absorbed dose). The cryotubes containing the irradiated cells were transported on dry ice, then stored in a −150 °C freezer, and analyzed.

### 4.4. RNA Sequencing Analyses

Frozen mouse ES cells were thawed at 37 °C. After adding the warmed culture medium [19], the cells were inoculated on an 8-hole plastic dish and cultured for the experimentally determined time. The pre-culture cell samples and time-indicated samples were taken separately after rubbing with scrapers. The cell suspensions were removed and mixed with Zenoll solution and gently mixed. The samples were frozen at −20 °C. The total RNA was isolated using a TRIZOL reagent (Thermo Fisher Scientific, Waltham, MA, USA). Then, 500 ng total RNA was used for RNA sequencing and analyzed by Tsukuba i-Laboratory LLP (Tsukuba, Ibaraki, Japan). Sequencing libraries were prepared using NEBNext rRNA Depletion Kit and NEBNext Ultra RNA Library Prep Kit for Illumina (New England Biolabs, Ipswich, MA, USA). Paired-end sequencing for 2 × 36 base reads was performed with NextSeq500 (Illumina, San Diego, CA, USA). FASTQ files were imported to CLC Genomics Workbench (CLC-WG, ver. 10.1.1, Qiagen, Hilden, Germany) for analysis. Expression values were calculated as “reads per kilobase per million reads (RPKM)” using the RNAseq analysis tool of CLC-GW. RPKM values were normalized by the quantile method. Fold change and difference were calculated for filtering differentially expressed genes. The data set was derived from a single space experiment, so the error bars and *p*-values are not shown.

## Figures and Tables

**Table 1:** Enhancement of gene expression by ionizing radiation.

ES Cell	C57BL6/129 Sv	B6-G	
Genotypes	H2AX (+/+)	H2AX (+/+)	H2AX (+/+)
Comparison	ISS/BU	Fe; 3 Gy/0 Gy	Fe; 3 Gy/0 Gy
Duration	0 h–8 h	0 h–8 h	0 h–48 h
Trp53 (p53)	0.94	0.98	1.09
Trp53inp1	1.53	1.33	1.45
Cdkn1a (p21)	1.13	1.28	1.34
Mdm2	1.23	1.20	1.35

## Data Availability

Data are contained within the article and Supplementary Materials.

## Supplementary Materials

The following supporting information can be downloaded at: https://www.mdpi.com/article/10.3390/ijms25063283/s1. References [87,88,89,90,91,92,93,94,95,96,97] are cited in the supplementary files.
