# Supplementary material for: Transcriptome Analysis by RNA Sequencing of Mouse Embryonic Stem Cells Stocked on International Space Station for 1584 Days in Frozen State after Culture on the Ground
Source: Int J Mol Sci. 2024 Mar 14;25(6):3283. doi: 10.3390/ijms25063283 (PMC10970647; doi:10.3390/ijms25063283)
Supplement: Supplementary file 1 [file ijms-25-03283-s001.zip › ijms-2868915-Supplementary Figures.pdf]

International Journal of
Molecular Sciences
MDPI

Supplementary Materials

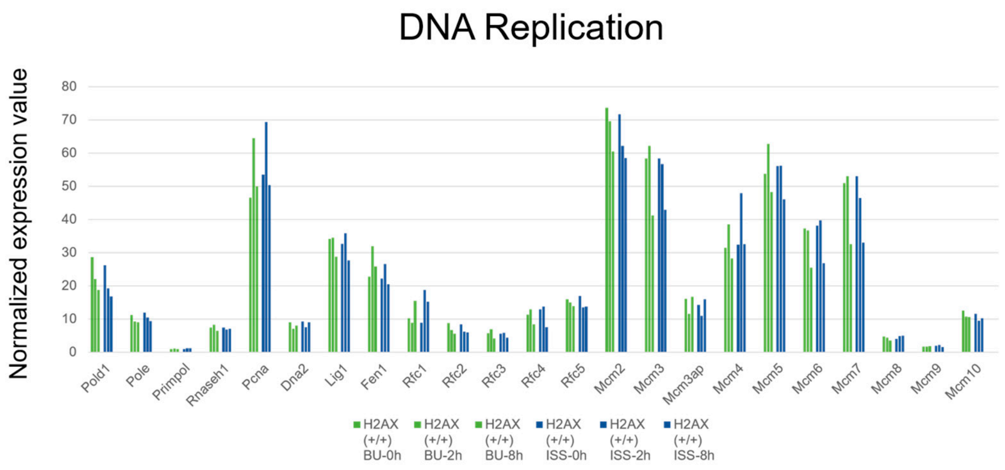

Figure S1. Expressions of genes involved in DNA replication in wild-type mouse ES cells on the ISS and on the ground.

The RNA levels of the respective gene in wild-type mouse ES cells are depicted. Blue bars represent the values of cells stocked on the ISS (ISS), while green bars represent ground backup controls (BU). They are arranged chronologically as 0, 2 and 8 hours after culture of the cells from right to left. The vertical axis displays the normalized expression values.

In DNA replication, the MCM complex opens the double strand DNA as a helicase to form a replication fork. Replication is initiated by the RNA primer synthesis by primase, PRIMPOL. The RNA primers are elongated by DNA POLδ and POLε. Then, the primer RNA is degraded by RNAaseH or peeled off by FEN1. The PCNA loaded by the RFC complex acts as a clamp to support the DNA polymerase to attach the DNA. LIG1 joins DNA that is synthesized discontinuously [87–89]. In this experiment, Mcm2-8 and Pcna genes expressed at high levels. In contrast, Primpol's expression was low. There was no discernible difference between ISS and BU in particular gene.

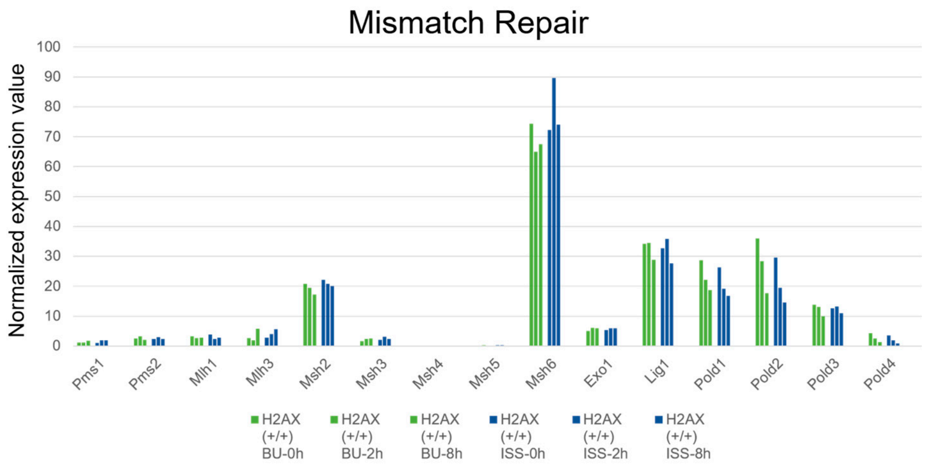

Figure S2. Expressions of genes involved in mismatch repair in wild-type mouse ES cells on the ISS and on the ground.

The RNA levels of the respective gene in wild-type mouse ES cells are depicted. Blue bars represent the values of cells stocked on the ISS (ISS), while green bars represent the ground backup controls (BU). They are arranged chronologically as 0, 2 and 8 hours after culture of the cells from right to left. The vertical axis displays the normalized expression values.

Mismatch repair is the process of repairing base mistakes, duplications and deletions in repeat sequences that occur in newly synthesized strands after DNA replication. Mismatches are recognized by complexes such as MSH2/MSH6. Then, the endonuclease PMS binds there, and removes mismatched bases from the nascent chain together with EXOI. After that, repair synthesis is carried out by Polδ and Polε, and the DNA is ligated by LIG1 [90]. Our result showed that Msh2 and Msh6 genes were highly expressed, but the RNA amounts of Mlh and Pms genes were relatively small. The ISS and BU samples showed no difference in the expression of genes related to mismatch repair.

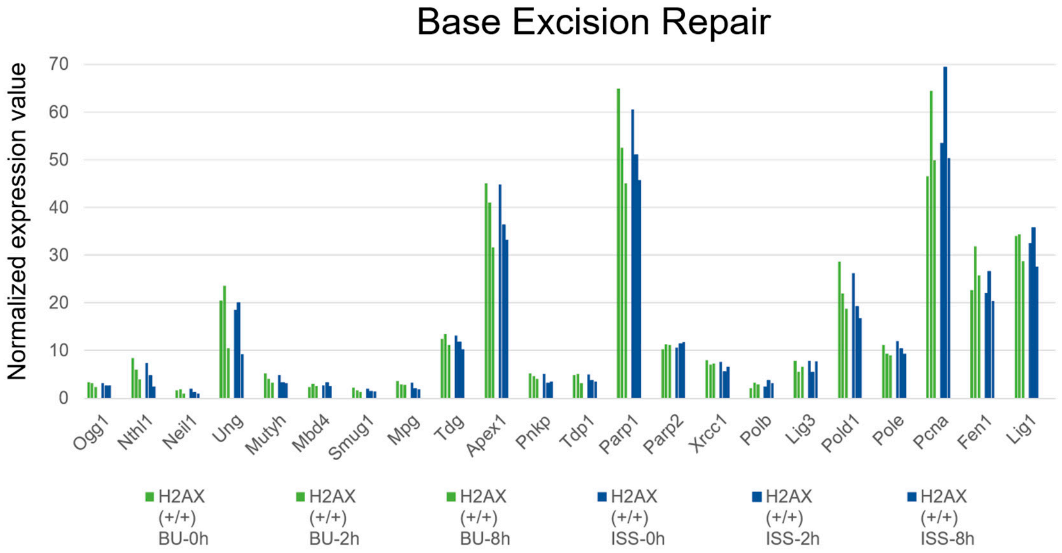

Figure S3. Expressions of genes involved in base excision repair in wild-type mouse ES cells on the ISS and on the ground.

The RNA levels of the respective gene in wild-type mouse ES cells are depicted. Blue bars represent the values of cells stocked on the ISS (ISS), while green bars represent the ground backup controls (BU). They are arranged chronologically as 0, 2 and 8 hours after culture of the cells from right to left. The vertical axis displays the normalized expression values.

Chemical modification of bases caused by environmental changes or metabolism results in mutations. Uracil DNA Glycosylase (UNG), MUTYH (MutY homolog) are glycosidases those remove modified bases. After removal, the AP endonuclease (APEX) excises the nucleotide, and XRCC1, LIGIII, POLβ, PARP, etc. form a complex to repair it. [91,92] Among these, the expression of Parp1, 2 and Apex1 genes were relatively strong. As the expression of Ung gene was relatively high, it was supposed that uracil would be abundant due to the deamination of cytosine. There were no obvious differences in the expression of these genes between ISS and BU.

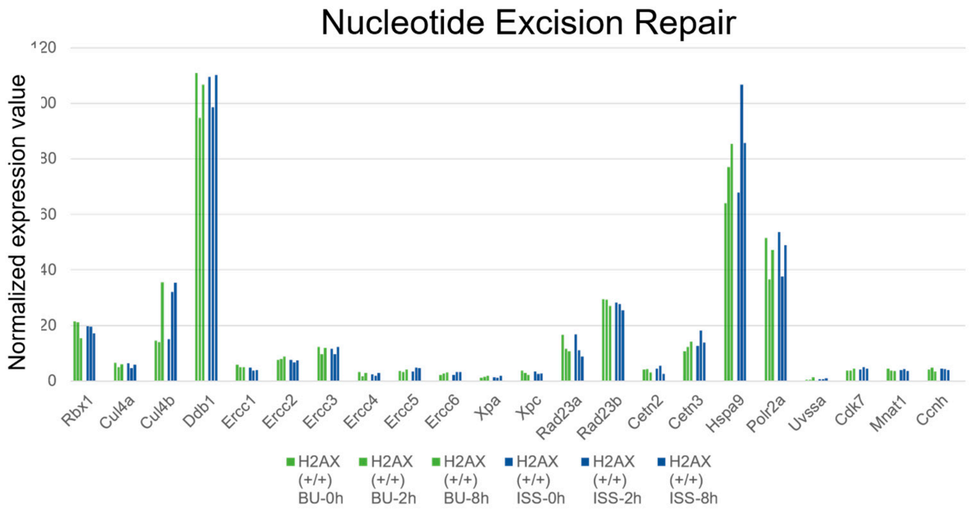

Figure S4. Expressions of genes involved in nucleotide excision repair in wild-type mouse ES cells on the ISS and on the ground.

The RNA levels of the respective gene in wild-type mouse ES cells are depicted. Blue bars represent the values of cells stocked on the ISS (ISS), while green bars represent the ground backup controls (BU). They are arranged chronologically as 0, 2 and 8 hours after culture of the cells from right to left. The vertical axis displays the normalized expression values.

Nucleotide excision repair is capable of repairing relatively long regions of damage, like thymine dimers that result from ultraviolet irradiation. Damaged DNA binding factor (DDB) recognizes and binds to UV damage site and is involved in the ubiquitin ligase reaction with CUL4. Next, XP proteins, which are causative for xeroderma pigmentosum, form the XPC complex. The Rbx1 gene and Rad23 gene are also required for nucleotide excision repair through ubiquitination. Repair synthesis occurs by PCNA, RFC, DNA POL δ/ε, etc., and the DNA ends are ligated by LIG1 [93–95]. The Ddb gene, necessary to identifies damaged sites, expressed at high level. There was no difference in gene expression between ISS and BU.

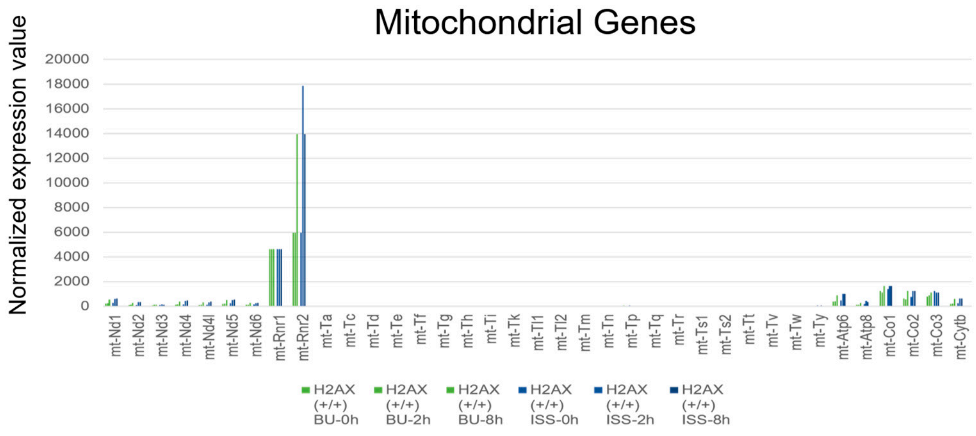

Figure S5. Expressions of mitochondrial genes in wild-type mouse ES cells on the ISS and on the ground.

The RNA levels of the respective gene in wild-type mouse ES cells are depicted. Blue bars represent the values of cells stocked on the ISS (ISS), while green bars represent the ground backup controls (BU). They are arranged chronologically as 0, 2 and 8 hours after culture of the cells from right to left. The vertical axis displays the normalized expression values.

Significant differences were observed in the expression of mitochondrial genes [96,97]. The expression of the NADH dehydrogenase genes (Mt-Nd) were low. A quite high expression of rRNA genes (Mt-Rnr1, 2) was observed. Cytochrome oxidase 1,2,3 and Cytochrome b genes expressed. Most genes of mitochondria were expressed at the same levels between ISS and BU, but there was a slight increase in the Rnr2 gene in ISS.
